# Prevalence, risk factors and clinical characteristics of renal dysfunction in Chinese outpatients with growth simple renal cysts

**DOI:** 10.1007/s11255-021-03065-5

**Published:** 2021-11-22

**Authors:** Qiaoru Wu, Chunhua Ju, Miaowen Deng, Xiaolong Liu, Zhongda Jin

**Affiliations:** 1 Department of Nephrology, Dongzhimen Hospital, Beijing University of Chinese Medicine, Beijing, 100700 China; 2 Department of Nephrology, Guangdong Provincial Hospital of Chinese Medicine, The Second Affiliated Hospital of Guangzhou University of Chinese Medicine, 111 Dade Road, Yuexiu District, Guangzhou, 510120 Guangdong Province China; 3 The Second Clinical College of Guangzhou, University of Chinese Medicine, Guangzhou, 510405 Guangdong Province China

**Keywords:** Growth simple renal cysts, Renal dysfunction, Prevalence, Risk factors, Clinical characteristics

## Abstract

**Background:**

Researchers have proved that simple renal cysts (SRCs) might be correlated with renal dysfunction, but it is still controversial. Thus, we conducted clinical research study with large sample size and long-term follow-up to clarify the relationship between SRCs and renal dysfunction.

**Methods:**

A total of 571 SRCs patients in outpatients of nephrology department were included, we investigated the clinical characteristics of growth SRCs compared with non-growth SRCs, evaluated the incidence of renal dysfunction in SRCs and explored the risk factors of renal dysfunction in growth SRCs.

**Results:**

The mean baseline age was 51.31 ± 14.37 years in the whole cohort, ranging from 19 to 79 years, and 57.6% of them were male. The median follow-up duration was 3 years, ranging from 1 to 10 years. In addition, the final maximum diameter increased 1 mm (2.74%) per year. Patients in growth SRCs group tented to have higher percentage of hypertension, hematuria, large cyst and multiple cysts compared with non-growth SRCs group. The prevalence of renal dysfunction was 15.6% after the follow-up, and the prevalence of renal dysfunction was about 10 times higher in growth SRCs group than non-growth SRCs group (23.3% vs*.* 2.4%). Renal dysfunction was significantly associated with age, female, total cholesterol, diastolic blood pressure, final maximum diameter and yearly change in maximum diameter in growth SRCs.

**Conclusions:**

SRCs were closely related to the decline of renal function, we recommend close follow-up for growth SRCs.

## Introduction

Kidneys play a vital role in maintaining fluid, electrolyte, and acid–base balance. The loss of renal function could lead to internal environmental disorders and develop into cardiovascular events. Moreover, hypertension, obesity, diabetes and dyslipidemia might lead to the coexistence of cardiovascular disease and renal dysfunction [1]. Clinical studies have proved that simple renal cysts (SRCs) (Bosniak I) might be correlated with renal dysfunction. SRC patients showed higher incidence of proteinuria, increased serum creatinine and decreased estimated glomerular filtration rate (eGFR) [2–4], and patients with larger cysts tended to be associated with hypertension and rapid decline in renal function [2, 5].

SRCs are the most common renal cystic diseases in adults, the prevalence of SRCs was reported to be 7.2–10.5% in Chinese cohorts, and higher in males and elders [3, 4, 6]. In addition, SRCs might grow in size and number over time [4, 6, 7]. The etiology of SRCs has not been clearly clarified. Some researchers speculated SRCs originated from the diverticula on the distal or collecting tubules [8]. Some researchers conducted clinical studies with small sample size and long-term follow-up of SRCs, but seldom clarified the relationship between growth SRCs and renal dysfunction. Our study compared the clinical characteristics of growth SRCs with non-growth SRCs, including serum creatinine, blood pressure, blood lipid levels and so on. The incidence of renal dysfunction in SRCs and risk factors of renal dysfunction in growth SRCs were also investigated.

## Methods

### Participants and measurements

**Fig. 1:**
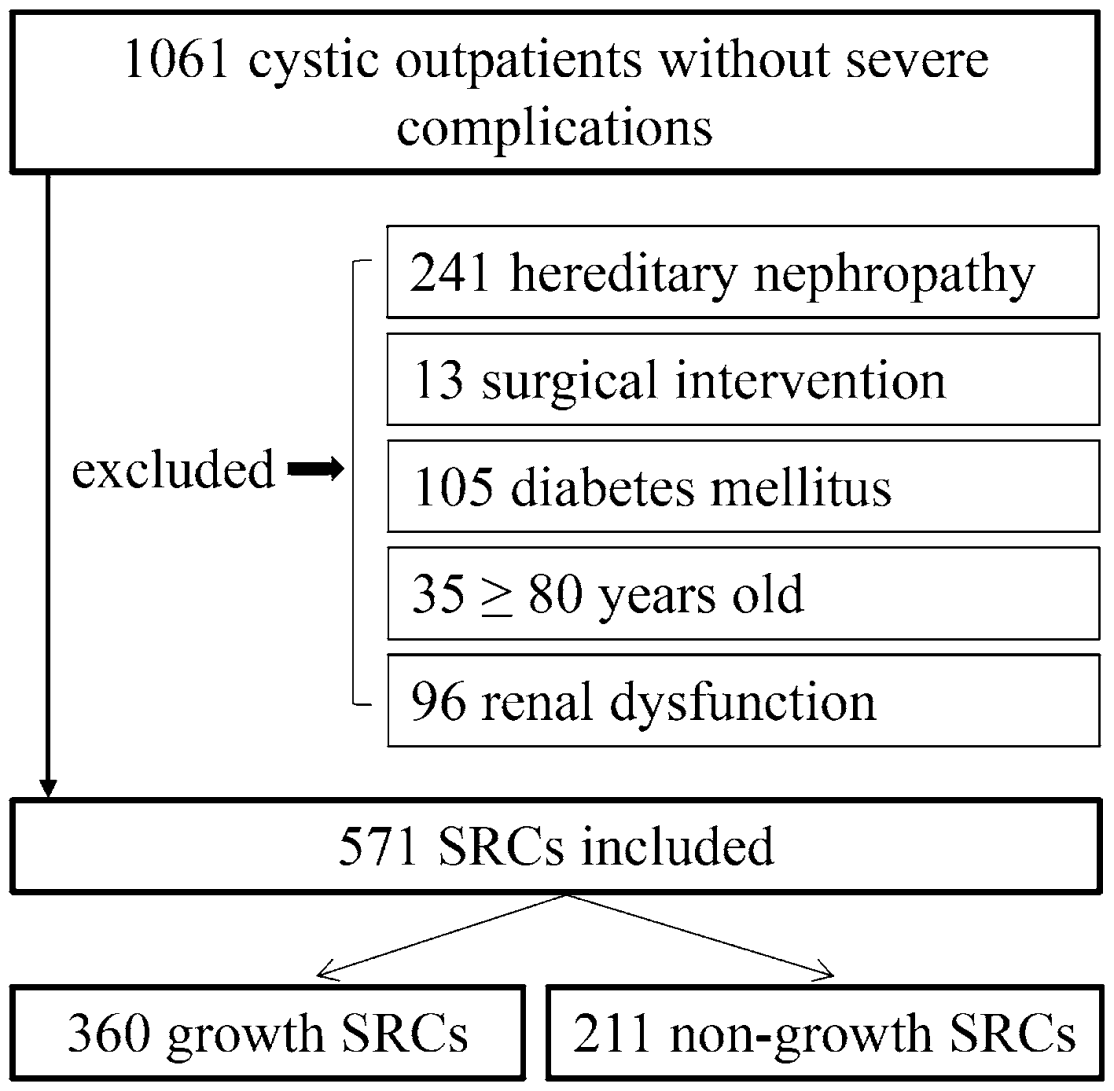
Details of the included patients

The current retrospective study was carried out in outpatients of nephrology department of the Second Affiliated Hospital of Guangzhou University of Chinese Medicine, from January 1st, 2010 to December 31st, 2019. We included patients meeting the following criteria: (1) 18–79 years old at the initial visit; (2) diagnosed with SRCs by ultrasound and reviewed at least once per year; (3) no surgical intervention; (4) no hereditary nephropathy, such as polycystic kidney disease and tuberous sclerosis; (5) no renal dysfunction, baseline eGFR > 60 mL/min/1.73 m^2^; (6) no severe complications, such as malignant diseases, autoimmune diseases, heart failure and severe liver diseases. We also excluded diabetes patients, because they might progress to diabetic nephropathy, which would significantly affect renal function. A total of 571 patients were included in the current study (Fig. 1).

The following data were collected: age, gender, systolic blood pressure (SBP), diastolic blood pressure (DBP), body mass index (BMI), follow-up period, complications (such as hypertension, hyperuricemia (HUA), hypercholesterolemia, hypertriglyceridemia, nephrolithiasis and hematuria) and laboratory examinations (serum creatinine, eGFR, serum uric acid (SUA), total cholesterol (TC), triglyceride (TG)). The ethics committee of Guangdong Provincial Hospital of Chinese Medicine approved the study (YE2019-197-01).

### Definition of SRCs, renal dysfunction, hypertension, hyperuricemia, dyslipidemia, hematuria and nephrolithiasis

SRCs were defined as a rounded or oval mass of homogeneous anechoic content, with marked posterior enhancement, smooth well-delimited margins, and an imperceptible sub-millimeter wall on ultrasound [9]. We defined multiple renal cysts for cyst number ≥ 2, large cysts for cyst diameter ≥ 20 mm, growth renal cysts for cyst diameter increased more than 5% of the basic or increased in number during the follow-up. Yearly change in maximum diameter of SRCs = (final maximum diameter—baseline maximum diameter)/follow-up duration. BMI was calculated as the weight (kg) divided by the square of the height (m^2^). According to MDRD formula, eGFR (mL/min/1.73 m^2^) = 175 × (serum creatinine/88.4)^−1.234^ × age^−0.179^ [× 0.79 (if female)] [10], we defined eGFR < 60 mL/min/1.73 m^2^ for renal dysfunction. Hypertension was defined as SBP ≥ 140 mmHg and/or DBP ≥ 90 mmHg, or by self-reported history of hypertension, or by the use of antihypertensives. Hyperuricemia was defined as SUA ≥ 420 μmol/L in male or ≥ 360 μmol/L in female, or by self-reported history of HUA, or by the use of urate-lowering therapy. Dyslipidemia was defined as total cholesterol ≥ 5.20 mmol/L and/or triglyceride ≥ 1.70 mmol/L, or by self-reported history of hypercholesterolemia or hypertriglyceridemia, or by the use of lipid-lowering drugs. Hematuria was defined as ≥ 3 red blood cells per high-powered field (HPF) by urine dipstick testing. Nephrolithiasis was defined as echogenic focus that with or without acoustic shadowing on ultrasound.

### Statistical analysis

Data were presented as mean ± standard or median (interquartile range) for continuous variables, and proportions for categorical variables. We used independent-samples *t* test or Mann*–*Whitney* U* test for continuous variables, and *χ*^*2*^ test for categorical variables to compare the differences in demographic and clinical variables between different groups. We used multivariable logistic regression analysis to estimate risk factors associated with renal dysfunction in growth SRCs group. *SPSS* version 23.0 was used for all statistical analysis with two tailed *P* values of 0.05. Kaplan*–*Meier and log-rank test were used for prevalence of renal dysfunction between growth SRCs group and non-growth SRCs group, using *GraphPad Prism* 8.0.2.

## Results

### Demographic characteristics among different groups

Among 571 patients, the mean baseline age was 51.31 ± 14.37 years, ranging from 19 to 79 years, and 329 (57.6%) of them were male. The median follow-up duration was 3 years, ranging from 1 to 10 years. Follow-up characteristics of the cohort stratified according to the development of SRCs are shown in Table 1. In the whole cohort, none of them progressed to a malignant cyst during the follow-up, and none of them decreased or disappeared, the final maximum diameter increased 1 mm (2.74%) per year. 353 patients increased in cyst size, 40 patients increased in cyst number, and 33 increased in both size and number. Finally, 360 patients were included in growth SRCs group. In growth SRCs group, the final maximum diameter increased 1.67 mm (6.25%) per year. In growth SRCs group, the mean age was 52.44 ± 14.42 years, while in non-growth SRCs group, the mean age was 58.06 ± 13.33 years. Patients in growth SRCs group tented to have higher percentage of hypertension (47.8% vs. 12.3%, *P* < 0.001), hematuria (24.7% vs. 13.3%, *P* = 0.001), large cyst (81.9% vs. 34.1%, *P* < 0.001) and multiple cysts (43.6% vs. 25.1%, *P* < 0.001) compared with non-growth SRCs group. Age, SBP, DBP and final maximum diameter were significantly different between growth SRCs group and non-growth SRCs group (all *P* < 0.001).

**Table 1:** The demographics and clinical characteristics of growth and non-growth SRCs

	All patients (*n* = 571)	Growth SRCs (*n* = 360)	Non-growth SRCs (*n* = 211)	^*^P value
Age, year				
Baseline	51.31 ± 14.37	48.98 ± 14.31	55.28 ± 13.62	< 0.001
Final	54.52 ± 14.27	52.44 ± 14.42	58.06 ± 13.33	< 0.001
Male, *n* (%)	329 (57.6)	196 (54.4)	133 (63.0)	0.045
BMI, kg/m^2^				
Baseline	22.57 ± 2.86	22.56 ± 3.09	22.59 ± 2.41	0.924
Final	22.65 ± 2.79	22.62 ± 2.99	22.69 ± 2.42	0.755
SBP, mmHg				
Baseline	130.38 ± 19.58	130.05 ± 19.46	130.94 ± 19.81	0.602
Final	132.15 ± 17.91	137.26 ± 18.36	123.44 ± 13.16	< 0.001
DBP, mmHg				
Baseline	79.04 ± 11.68	78.91 ± 11.86	79.25 ± 11.38	0.739
Final	79.71 ± 11.56	81.73 ± 12.25	76.26 ± 9.33	< 0.001
Scr, μmol/L				
Baseline	78.57 ± 16.23	77.50 ± 15.92	80.39 ± 16.62	0.039
Final	90.90 ± 20.80	97.39 ± 20.90	79.82 ± 15.24	< 0.001
eGFR, mL/min/1.73 m^2^				
Baseline	96.42 ± 23.63	98.10 ± 23.88	93.54 ± 22.97	0.026
Final	80.72 ± 23.08	73.47 ± 19.62	93.10 ± 23.32	< 0.001
SUA, μmol/L				
Baseline	366.25 ± 102.24	364.93 ± 103.53	368.52 ± 100.19	0.686
Final	360.78 ± 104.76	360.88 ± 105.62	360.61 ± 103.51	0.976
TC, mmol/L				
Baseline	5.06 ± 1.14	5.04 ± 1.13	5.10 ± 1.14	0.572
Final	5.30 ± 1.69	5.41 ± 1.66	5.13 ± 1.72	0.058
TG, mmol/L				
Baseline	1.19 (0.87, 1.74)	1.19 (0.86, 1.75)	1.18 (0.87, 1.70)	0.958
Final	1.32 (0.94, 1.82)	1.30 (0.92, 1.82)	1.34 (0.94, 1.84)	0.365
Hypertension, *n* (%)				
Baseline	209 (36.6)	140 (38.9)	69 (32.7)	0.138
Final	198 (34.7)	172 (47.8)	26 (12.3)	< 0.001
Renal dysfunction, *n* (%)				
Final	89 (15.6)	84 (23.3)	5 (2.4)	< 0.001
Hyperuricemia, *n* (%)				
Baseline	193 (33.8)	128 (35.6)	65 (30.8)	0.247
Final	204 (35.7)	132 (36.7)	72 (34.1)	0.540
Hypercholesterolemia, *n* (%)				
Baseline	248 (43.4)	155 (43.1)	93 (44.1)	0.812
Final	289 (50.6)	194 (53.9)	95 (45.0)	0.041
Hypertriglyceridemia, *n* (%)				
Baseline	149 (26.1)	96 (26.7)	53 (25.1)	0.684
Final	149 (26.1)	113 (31.4)	63 (29.9)	0.702
Nephrolithiasis, *n* (%)				
Baseline	180 (31.5)	113 (31.4)	67 (31.8)	0.928
Final	185 (32.4)	118 (32.8)	67 (31.8)	0.801
Hematuria, *n* (%)				
Baseline	93 (16.3)	66 (18.3)	27 (12.8)	0.084
Final	117 (20.5)	89 (24.7)	28 (13.3)	0.001
Maximum diameter, mm				
Baseline	21.00 (14.00, 38.00)	26.50 (16.00, 43.00)	17.00 (12.00, 23.00)	< 0.001
Final	25.00 (16.00, 44.00)	34.00 (23.00, 51.75)	17.00 (12.00, 23.00)	< 0.001
Yearly change in maximum diameter, mm/year	1.00 (0.00, 2.00)	1.67 (1.00,2.50)	–	–
Large cyst, *n* (%)				
Baseline	316 (55.3)	244 (67.8)	72 (34.1)	< 0.001
Final	367 (64.3)	295 (81.9)	72 (34.1)	< 0.001
Multiple renal cysts, *n* (%)				
Baseline	170 (29.8)	117 (32.5)	53 (25.1)	0.063
Final	210 (36.8)	157 (43.6)	53 (25.1)	< 0.001

*BMI* body mass index; *SBP* systolic blood pressure; *DBP* diastolic blood pressure; *Scr* serum creatinine; *eGFR* estimated glomerular filtration rate; *SUA* serum uric acid; *TC* total cholesterol; *TG* triglyceride

^*^Compared growth SRCs with non-growth SRCs

### Prevalence of renal dysfunction in SRCs

**Fig. 2:**
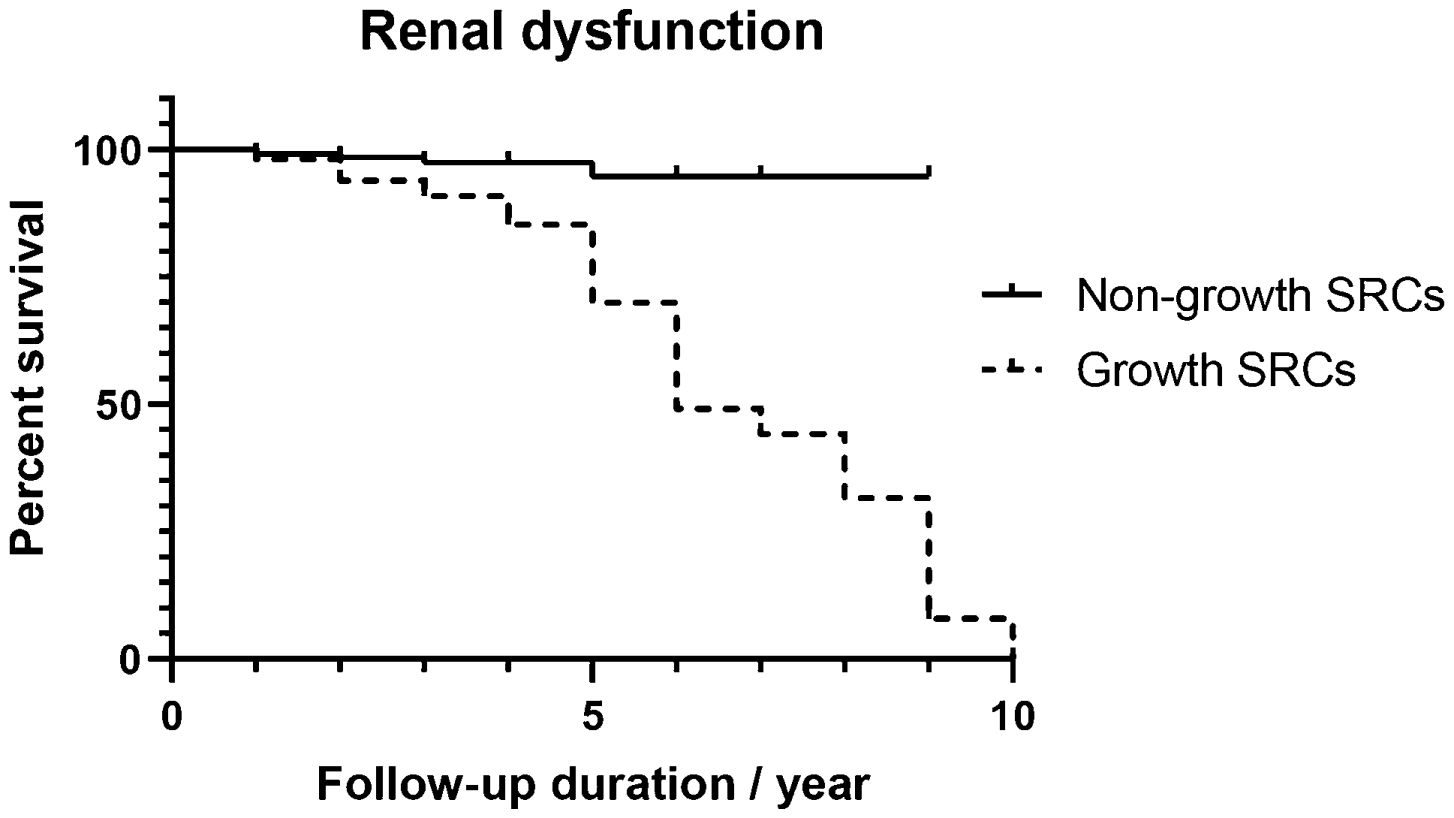
The K–M curve for the free survival of renal dysfunction between growth SRCs and non-growth SRCs (log-rank* P* < 0.001)

The mean baseline serum creatinine was 78.57 ± 16.23 μmol/L, and the mean baseline eGFR was 96.42 ± 23.63 mL/min/1.73 m^2^ in the whole cohort. As shown in Table 1, the prevalence of renal dysfunction was 15.6% (89/571) after the follow-up. The prevalence of renal dysfunction was 23.3% in growth SRCs group and 2.4% in non-growth one, with a significant difference between them (*P* < 0.001). The mean serum creatinine was 97.39 ± 20.90 μmol/L in growth SRCs group, and 79.82 ± 15.24 μmol/L in non-growth SRCs group. The mean eGFR was 73.47 ± 19.62 mL/min/1.73 m^2^ in growth SRCs group, and 93.10 ± 23.32 mL/min/1.73 m^2^ in non-growth SRCs group. Serum creatinine and eGFR were significantly different between growth SRCs group and non-growth SRCs group (both *P* < 0.001). The probability of free survival of renal dysfunction based on the development of SRCs in the Kaplan*–*Meier analysis showed significant differences, as shown in Fig. 2.

### Demographic and clinical characteristics between renal dysfunction and normal renal function in growth SRCs

As shown in Table 2, growth SRCs patients with renal dysfunction were more likely to be older (57.67 ± 13.36 vs. 50.85 ± 14.38 years old), female (60.7% vs. 40.9%), hypertension (71.4% vs. 40.6%), HUA (56.0% vs. 30.8%), hypercholesterolemia (78.6% vs. 46.4%), hypertriglyceridemia (44.0% vs. 27.5%) and large cyst (97.6% vs. 77.2%), with higher levels of SBP (146.46 ± 18.75 vs. 134.46 ± 17.33 mmHg), DBP (87.49 ± 11.40 vs. 79.98 ± 11.98 mmHg), SUA (386.23 ± 107.15 vs. 353.16 ± 104.13 μmol/L), TC (6.39 ± 1.53 vs. 5.11 ± 1.59 mmol/L), TG (1.63 vs. 1.24 mmol/L), larger final maximum diameter (49 vs. 31 mm) and larger yearly change in maximum diameter (2.16 vs. 1.50 mm/year), with significant differences compared with normal renal function group (all *P* < 0.05). After adjusted for the confounding factors, using multivariable logistic regression analysis, renal dysfunction was associated with age (OR 1.031, 95% CI 1.008–1.055), female (OR 3.739, 95% CI 2.002–6.983), TC (OR 1.574, 95% CI 1.301–1.905), DBP (OR 1.036, 95% CI 1.008–1.065), final maximum diameter (OR 1.017, 95% CI 1.002–1.032) and yearly change in maximum diameter (OR 1.238, 95% CI 1.048–1.462) (Table 3).

**Table 2:** Demographics and clinical characteristics between renal dysfunction and normal renal function in growth SRCs

	Renal dysfunction (*n* = 84)	Normal renal function (*n* = 276)	*P* value
Age, year	57.67 ± 13.36	50.85 ± 14.38	< 0.001
Male, *n* (%)	33 (39.3)	163 (59.1)	0.001
BMI, kg/m^2^	22.08 ± 2.48	22.79 ± 3.11	0.056
SBP, mmHg	146.46 ± 18.75	134.46 ± 17.33	< 0.001
DBP, mmHg	87.49 ± 11.40	79.98 ± 11.98	< 0.001
SUA, μmol/L	386.23 ± 107.15	353.16 ± 104.13	0.012
TC, mmol/L	6.39 ± 1.53	5.11 ± 1.59	< 0.001
TG, mmol/L	1.63 (1.00, 1.90)	1.24 (0.91, 1.80)	0.011
Hypertension, *n* (%)	60 (71.4)	112 (40.6)	< 0.001
Hyperuricemia, *n* (%)	47 (56.0)	85 (30.8)	< 0.001
Hypercholesterolemia, *n* (%)	66 (78.6)	128 (46.4)	< 0.001
Hypertriglyceridemia, *n* (%)	37 (44.0)	76 (27.5)	0.004
Nephrolithiasis, *n* (%)	24 (28.6)	94 (34.1)	0.348
Hematuria, *n* (%)	25 (29.8)	64 (23.2)	0.221
Final maximum diameter, mm	49.00 (35.25, 65.00)	31.00 (21.00, 47.00)	< 0.001
Yearly change in maximum diameter, mm/year	2.16 (1.50, 3.40)	1.50 (1.00, 2.50)	< 0.001
Large cyst, *n* (%)	82 (97.6)	213 (77.2)	< 0.001
Multiple renal cysts, *n* (%)	39 (46.4)	118 (42.8)	0.552

**Table 3:** Multivariable logistic regression analysis for association between demographics and renal dysfunction in growth SRCs

	^a^Adjusted OR (95% CI)	*P* value
Age, per 1 year increase	1.031 (1.008–1.055)	0.008
Gender, female vs*.* male	3.739 (2.002–6.983)	< 0.001
TC, per 1 mmol/L increase	1.574 (1.301–1.905)	< 0.001
DBP, per 1 mmHg increase	1.036 (1.008–1.065)	0.012
Large cyst, positive vs*.* negative	4.488 (0.949–21.219)	0.058
Final maximum diameter, per 1 mm increase	1.017 (1.002–1.032)	0.029
Yearly change in maximum diameter, per 1 mm/year increase	1.238 (1.048–1.462)	0.012

^a^Adjusted for age, gender, SBP, DBP, SUA, TC, TG, hypertension, hyperuricemia, hypercholesterolemia, hypertriglyceridemia, final maximum diameter, yearly change in maximum diameter and large cyst

## Discussion

To our best knowledge, the current study had the largest SRC sample size in Chinese outpatients. We found higher rate of renal dysfunction compared with another Chinese research after follow-up (15.6% vs. 3.8%) [2]. Such difference may be related to the source of patients. In our study, all patients visited nephrology outpatient department rather than health examination center. It is worth noting that the prevalence of renal dysfunction was about 10 times higher in growth SRCs group compared with non-growth SRCs group in our cohort study participants (23.3% vs. 2.4%).

The reliability of diagnosing SRCs by ultrasound is approximately 100% [9], the current study only used ultrasound results to evaluate the development of SRCs. But the measurement of the maximum diameter of SRCs by ultrasound has the intrapersonal or interpersonal variability. The limitation of measurement for the diameter of SRCs by ultrasound exists, and therefore, the judgment of growth SRCs also has uncertainty. In general, SRCs are asymptomatic, they rarely progress to malignant cysts [6, 7, 11]. However, more than half of our patients complained of low back pain or hematuria in varying degrees. Growth SRCs showed higher proportion of hematuria than non-growth SRCs after follow-up (24.7% vs. 13.3%). Large cysts (81.9% vs. 34.1%) and multiple cysts (43.6% vs. 25.1%) were more common in growth SRCs compared with non-growth SRCs. In growth SRCs group, the final maximum diameter increased 1.67 mm (6.25%) per year. None of SRCs progressed to a malignant cyst during the follow-up. SRCs were more common in males [3, 4, 6]. In the current cohort, 57.6% of the patients were male. Liu et al. [12] examined the expression of signal transducer and activator of transcription 3 (STAT3) and androgen receptor in cystic kidneys and normal kidneys, they found strongly activated STAT3 and positive androgen receptor in tubular epithelial cells from cystic kidneys, but rare in normal kidneys. Therefore, they hypothesized that the gender disparity in SRCs might be related to androgen-STAT3 activation. Contrary to expectations, in growth SRCs group, renal dysfunction was significantly related to female (OR 3.739, 95% CI 2.002–6.983). Further hormone examinations are needed to explain the relationship between gender and renal dysfunction in SRCs. The prevalence of SRCs increases with age [4, 6, 13], 56.9% of our patients were older than 50 years at the initial visit, the age-related incidence might be relevant to the increasing diverticula of renal tubules with aging [14]. It is remarkable that patients in growth SRCs group were younger than non-growth SRCs group (52.44 ± 14.42 vs. 58.06 ± 13.33 years). Similarly, Park et al. [7] found the probability of an increase in SRCs was 7.1 times greater in patients ≥ 50 years old at diagnosis than in those < 50 years old, but the mean growth rate was lower in patients ≥ 50 years old.

Kidney cortical volume would decrease and the number and size of SRCs would increase with healthy aging, accompanied with decline of nephron number and decreased renal function [14]. Therefore, the decline in renal function whether caused by SRCs is still controversial. Kwon et al. [5] used dimercaptosuccinic acid renal scans to examine cystic kidney function compared with the contralateral normal kidney. They found the relative renal function values of the cystic kidney were significantly lower than the contralateral kidney in SRCs group, but there were no differences between two kidneys in normal kidney group. Another research investigated the effect of cysts on living renal transplantation function [15]. It found allograft functions were both normal in cyst group and non-cyst group, but cysts in donor kidney could affect allograft function, and cystic donors had more sclerotic glomeruli than non-cyst group. Other researches declared that SRCs were associated with higher incidence of proteinuria, increased serum creatinine and decreased eGFR [2–4]. All the above showed negative effects of SRCs on renal function. However, Ozdemir et al. [16] suggested that SRCs had no effect on renal dysfunction. Waldram et al. [17] found kidneys with cysts had lower baseline eGFR but similar yearly change in eGFR compared to those without cysts. The creatinine credibility as endogenous biomarker is also exposed in recent conducted research studies as standard eGFR marker [18, 19]. In our study, the mean serum creatinine of the whole cohort after follow-up was significantly higher than the baseline level (90.90 ± 20.80 vs. 78.57 ± 16.23 μmol/L), the mean serum creatinine of growth SRCs group was significantly higher than non-growth SRCs group after follow-up, and the mean eGFR of growth SRCs group was significantly lower than non-growth SRCs group. Our results showed that renal dysfunction was associated with final maximum diameter and yearly change in maximum diameter in growth SRCs group, therefore, we approved that cyst expansion further aggravated the decline of nephron number, resulted in the rapid decline in renal function.

There was a significantly increased proportion of hypertension in growth SRCs, and renal dysfunction was correlated with blood pressure level in our study. Kim et al. [20] analyzed the relationship between newly SRCs and hypertension, they found SRCs, especially bilateral cysts, multiple cysts, and cysts that maximum diameter larger than 1 cm, were significantly positively related to the incidence of hypertension. Similarly, Lee et al. [21] found the presence of SRCs increased SBP by 2.38 mmHg, increased DBP by 1.61 mmHg, and multiple cysts and large cysts were closely related to prehypertension and hypertension in adults. Some reports presented that the surgical intervention of SRCs resulted in a reduction of blood pressure [22, 23]. Another research indicated that SRCs were associated with increased arterial stiffness [24]. Clinically, arterial stiffness is measured through simultaneous oscillometric measurement of the brachial and ankle pulse wave velocity (baPWV). Wu et al. [24] found SRCs could increase the values of baPWV, and both the size and number of SRCs were positively correlated with an increased value of baPWV. The interaction of hypertension and SRCs is still unclear. It is reported that SRCs might compress renal artery, or cystic expansion lead to renal ischemia, and then activate the renin-angiotensin system, thus result in hypertension [20]. We also discovered that SUA was associated with renal dysfunction in growth SRCs, patients in renal dysfunction group showed higher percentage of HUA compared with normal renal function group. However, these relationships disappeared after adjusted for the confounding factors. Similar to our results, Kwon et al. [5] reported that the decrease in renal function in SRCs was associated with higher SUA levels, they explained that SRCs caused renal dysfunction first and elevated SUA later, and our results seemed to support this hypothesis.

The subjects of the study were all outpatients from nephrology department, which may lead to unavoidable selection bias. In addition, the current retrospective study did not exclude the effects of drugs on the growth of SRCs and it could not express the natural process of SRCs. The existence of SRCs was often overlooked due to its rare progression to significant complications. However, our research found that SRCs were closely related to the decline of renal function and the prevalence of renal dysfunction was about 10 times higher in growth SRCs group than non-growth SRCs group. We recommend close follow-up for growth SRCs.
